# The acute effects of continuous and intermittent whole-body passive heating on cardiovascular disease risk indicators in healthy and young males and females

**DOI:** 10.1007/s00421-025-05718-0

**Published:** 2025-02-14

**Authors:** Yunuo Su, Adela Martinkova, Emma O’Donnell, Stephen J. Bailey, Christof A. Leicht

**Affiliations:** 1 Peter Harrison Centre for Disability Sport, School of Sport, Exercise and Health Sciences, Loughborough University, Loughborough, LE11 3TU UK; 2 School of Sport, Exercise and Health Sciences, Loughborough University, Loughborough, LE11 3TU UK

**Keywords:** Skin perfusion, Arterial stiffness, Inflammation, Body temperature, Thermal comfort, Passive heat

## Abstract

**Purpose:**

Heat therapy is recognised to promote cardiovascular health, and whilst most recent heat therapy investigations have focussed on continuous heat exposure, traditional sauna use often includes recovery periods. This study compared the acute effects of continuous versus intermittent whole-body heating on cardiovascular function markers in males and females.

**Methods:**

Twenty healthy participants (25 ± 3 years; 10 males, 10 females) were exposed to 2 passive heating regimens: continuous heating (CH) for 60 min and intermittent heating (IH) comprised of 3 × 20-min blocks interspersed by 15-min cooling breaks. Skin perfusion, blood pressure (BP), plasma nitrite, interleukins, body temperature, and thermal perceptual responses were assessed.

**Results:**

Greater increases in rectal temperature (T_rec_) (CH: 1.2 ± 0.1 °C; IH: 0.5 ± 0.1 °C), skin perfusion, systolic blood pressure (SBP), heart rate (HR), interleukin-6 (IL-6) and plasma nitrite were found in CH compared to IH (*p* ≤ 0.01), but the thermal perceptual response was more unfavourable during CH (*p* < 0.01). Females had higher skin perfusion and plasma nitrite concentrations (*p* ≤ 0.04), but lower brachial and central BP than males in both conditions (*p* ≤ 0.01). Furthermore, females reached a higher T_rec_ and more unfavourable thermal perception in CH (*p* ≤ 0.02).

**Conclusion:**

More pronounced cardiovascular responses were associated with higher T_rec_ and discomfort. Females exhibited higher skin perfusion and plasma nitrite concentrations than males and reported less favourable thermal perception in CH, but not in IH.

## Introduction

Passive heating acutely affects the cardiovascular system, inducing an increase in cardiac output, arterial blood flow, shear stress (Chiesa et al. 2015) and blood pressure (Hoekstra et al. 2018). Heat therapy is hence emerging as a potential alternative to exercise for improving cardiovascular health, by improving various vascular properties including microvascular function, endothelial function and arterial stiffness (Cheng and MacDonald 2019). The improvements in endothelial function (Laughlin et al. 2008) can be partly explained by the heat-induced acute elevations of plasma nitrite concentrations (Hoekstra et al. 2021), a proxy marker of endothelium-derived nitric oxide (NO) (Lauer et al. 2001), which is instrumental for vasodilation, facilitating increased blood flow (Harrison et al. 2006). These improvements in cardiovascular disease risk factors may help explain why repeated exposure to passive heat stress can reduce arterial stiffness, evidenced by increases in superficial femoral arterial compliance, and decreased femoral β-stiffness index and aortic pulse wave velocity (Brunt et al. 2016b). It further helps to reinforce epidemiological data indicating a relationship between passive heat exposure (e.g. Sauna bathing and Tub bathing) and reduced cardiovascular disease (CVD) morbidity and mortality (Laukkanen et al. 2015; Ukai et al. 2020).

In addition, chronic passive heat exposure has been linked to improvements in chronic low-grade inflammation (Hoekstra et al. 2018), which is known to play a crucial role in the development of CVD (Okazaki et al. 2014; Held et al. 2017). This improvement in inflammation may result from the anti-inflammatory response following the IL-6 acute increase induced by passively raising T_rec_ by approximately 1.0–2.0℃ (Hoekstra et al. 2018; Su et al. 2024). Indeed, IL-6 may counteract chronic low-grade inflammation by stimulating the production of anti-inflammatory cytokines such as interleukin-1 receptor antagonist (IL-1ra) and interleukin-10 (IL-10) (Petersen and Pedersen 2005). There may be sex differences in this inflammatory response. For example, a higher IL-6 response in females than in males has been observed 60 min after maximal intensity cycling tasks (Edwards et al. 2006). On the other hand, other studies report that the acute resistance exercise-induced IL-6 response has been found to be smaller in women compared to men (Aragón-Vela et al. 2021). The reasons for these apparently conflicting findings are unknown. However, in vitro studies demonstrate that oestrogen inhibits IL-6 gene expression by impairing the transactivation of nuclear factor κB (Liu et al. 2005). In premenopausal women, IL-6 has been reported to be elevated during the low versus high oestrogen phases of the menstrual cycle (Eagan et al. 2021). However, sex differences in the inflammatory response to heating largely remain unknown.

Studies documenting acute cardiovascular and inflammatory changes during passive heating often use a pronounced heat stimulus, with 60-min exposures resulting in substantial increases in T_rec_ (~ 38.5 °C) (Brunt et al. 2016b; Hoekstra et al. 2018), and as a result, a negative perceptual response (Hoekstra et al. 2018). Nevertheless, smaller improvements in arterial endothelial function were observed using less intense heating, such as shorter durations (Carter et al. 2014) and smaller core temperature increases (Bailey et al. 2016). Similarly, despite a mild passive heating stimulus resulting in modest core temperature elevations (~ 0.6 ℃), an inflammatory response was observed (Kaldur et al. 2016; Hoekstra et al. 2021).

A typical duration of sauna bathing can vary between 5 and 30 min and can include short recovery periods in a colder environment (Heinonen and Laukkanen 2018). It has been mentioned previously that little is known about the ideal frequency, temperature, and duration of heat therapy (Cheng and MacDonald 2019), and the investigation of a heat exposure pattern, which is broken up with cooling periods, remains to be explored. In addition, most studies have overlooked potential sex differences in thermal perception (Jung et al 2014; Hoekstra et al. 2018), despite women consistently reporting higher levels of thermal discomfort than men at high ambient temperatures (Karjalainen 2007).

Therefore, the primary aim of this study was to investigate the effects of acute continuous heat exposure for 60 min or acute repeated heat exposure (3 bouts of 20 min, interspersed by breaks) on skin perfusion, blood pressure, pulse wave variables, plasma nitrite, IL-6, IL-1ra, and IL-10 and thermal perception. A secondary aim was to evaluate sex differences in these responses.

## Methods

Ten male and 11 female participants completed all experiments, for females, the main trials were scheduled in days 1–7 of their menstrual cycle. All female participants self-reported having regular menstrual cycles with durations ranging from 21 to 35 days. Data from one female participant were excluded due to her 17β-estradiol and progesterone levels exceeding the criteria for the early follicular phase (estradiol: > 144.1 pg/mL (NHS 2022); progesterone: > 1.6 ng/mL (NHS 2024)). Consequently, analyses were performed on data from ten males and ten females, all of whom were young, healthy, and non-smokers. Participants underwent health screening and were excluded if they had any history of cardiovascular-related conditions, used hormonal contraceptives, consumed anti-inflammatory medications, or engaged in self-reported structured physical activity exceeding 10 h weekly. The study was approved by the Loughborough University Ethics Committee (project code: 12,888), in accordance with the ethical standards outlined in the Declaration of Helsinki with the exception of registering in a public database.

### Study design

**Fig. 1:**
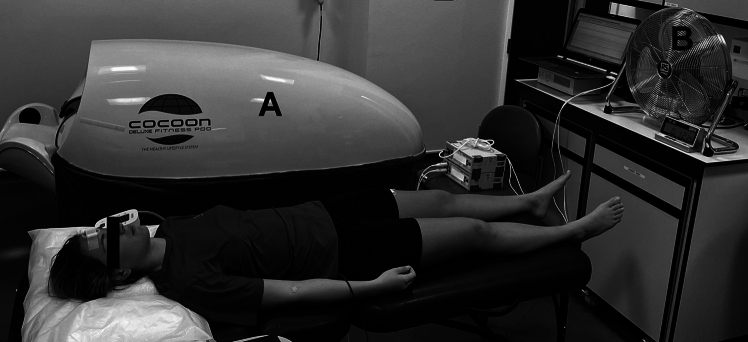
The setup of the experiment. **A** Cocoon POD heating device; **B** fan used for cooling in IH protocol

Participants attended baseline anthropometric measurements in the prior visit. Subsequently, laboratory testing was conducted on two main occasions, with visits separated by a minimum wash-out period of 2 days between main trials to minimise any influence from prior heating. Prior to each session, dietary intake and exercise were standardised by recording 24 h of food consumption for the first main trial and replicating this for the subsequent main trial, participants refrained from engaging in physical exercise and from consuming caffeine and alcohol. All sessions commenced between 11 am and 1 pm to minimise the impact of circadian variations on key outcome measures, such as body temperature. The experimental protocol comprised two passive heating conditions in the supine position, allocated in a random sequence determined through an online randomisation tool (https://randomizer.org/#randomize). One condition involved 60 min of continuous whole-body heating (CH), whereas the other consisted of three 20-min periods of heating, each separated by 15-min supine rest intervals, with a 10-min fan cooling period included during the rest phases (IH). To enhance perceived comfort and ensure completion of the session, a 20-min period of facial cooling was applied in the last 20 min of the 60-min heating phase in both conditions. Participants were allowed ad libitum water intake during the sessions. Heating was applied using a Cocoon POD heating device (Wellness USA, Minneapolis, MN, USA), which functions as a dry sauna with infrared light. This device exposed the body to temperatures of 71.6 ± 1.8 °C during CH and 71.9 ± 1.6 °C during IH (Fig. 1). To mitigate any potential variations in body temperature, thermal perception, or heat exposure as a result of clothing, participants wore a standard T-shirt and shorts made from 100% polyester for both main trials. During IH, whole-body cooling was applied by an 18-inch diameter High Velocity 3 Speed Floor Fan (RS PRO, Northants, UK). The fan was placed 50 cm from the participant, and the fan speed was 4.4 m/s (Speed setting 1) measured by a Kestrel 3000 Environmental Meter (Richard Paul Russell Ltd, Lymington, UK). The ambient temperature and relative humidity within the laboratory were 21.9 ± 0.6 °C and 41.7 ± 14.1% during CH, and 21.9 ± 0.7 °C and 40.9 ± 12.6% during IH.

### Procedures—preliminary visit

Prior to the main experimental sessions, each participant underwent a preliminary visit to the laboratory where anthropometric measures of height, body mass, body composition (seca mBCA 515, Hamburg, Germany), and skinfolds (biceps, triceps, supra-iliac and subscapular) for the estimation of body fat percentage (Durnin and Womersley 1974) were collected. 

### Procedures—main trials

Participants were required to complete the skin preparation for the laser Doppler flowmetry (LDF) (moorVMS-LDF, Moor Instruments, Devon, UK) measurement themselves by shaving the relevant areas 24 h prior to the test.

On arrival, participants applied a T_rec_ probe 10 cm beyond the anal sphincter for the measurement of T_rec_. Incremental area under curve (iAUC) for T_rec_ during heating was calculated using the trapezoidal method. Nude body mass was then assessed to the nearest 0.01 kg (Seca 284, Hamburg, Germany). Skin temperature (T_skin_) sensors (iButton, Homechip Ltd, Milton Keynes, UK) were fitted on the cheek, chest, arm, thigh, and calf using TransporeTM surgical tape (3M Healthcare, St Paul, Minnesota, USA). Measurements for T_skin_ were taken from the left side of the body, and the mean T_skin_ was calculated using the formula: Mean T_skin_ = 0.3*chest T_skin_ + 0.3*arm T_skin_ + 0.2*thigh T_skin_ + 0.2*calf T_skin_ (Ramanathan 1964).

Thereafter, participants rested supine for 30 min. During this rest period, the skin of the forearm and thigh was cleansed with distilled water. Absorbent patches (5 cm × 7 cm; 3M Tegaderm™ + Pad; absorbent pad size 4.3 cm × 2.8 cm) were applied to the mid-ventral forearm and mid-ventral thigh. The skin was dried immediately before applying the patches with non-sterile gauze swabs (Premier Healthcare & Hygiene Ltd, Gateshead, UK) on the mid-ventral forearm and mid-ventral thigh, and each patch was weighed before use. At the end of the rest period, the temperature measures, heart rate (HR; Polar, Kempele, Finland), blood pressure (Omron healthcare, Kyoto, Japan), and vascular function (pulse wave analysis using SphygmoCor, Atcor Medical, Sydney, Australia) metrics were recorded. Furthermore, participants reported their thermal sensation (Epstein and Moran 2006), thermal comfort (Gagge and Nishi 1976) and skin wetness (Filingeri et al. 2015), whilst basic emotions were assessed using the Feeling Scale (Hardy and Rejeski 1989). Blood samples were collected after completing the physiological and perceptual measurements.

Afterwards, participants entered the Cocoon POD for passive heating. Physiological and perceptual measurements were assessed every 20 min during CH, and after each 20-min heating block during IH. At the end the passive heating period, the absorbent patches were immediately removed using a clean forceps, placed in the previously used petri dish and weighed to determine local sweat loss. Blood samples were also collected at this time point via venipuncture of the antecubital vein. After that, the nude body was reassessed to estimate the amount of sweat loss, taking into account water intake. After the session, participants rested supine for an additional 60 min to further assess physiological and perceptual responses at 30 and 60 min post-intervention, followed by another blood sample collection.

### Pulse wave analysis

Pulse wave analysis was assessed at the radial artery using applanation tonometry methods to determine central BP and indices of arterial stiffness. Radial artery pressure waveforms, calibrated against brachial artery blood pressure readings, were utilised to derive the central aortic pressure waveform using a validated generalised transfer function (Chen et al. 1997). Pulse wave analysis indices of interest included aortic blood pressure components (central systolic, diastolic, mean arterial, and pulse pressure; central SBP, central DBP, central MAP, and central PP, respectively), and augmentation index adjusted for heart rate of 75 bpm (AIx@HR75) is reported.

Baseline measurements were taken after 20 min of supine rest. Post-intervention and 60-min recovery measurements were taken between 55 and 60 min of the intervention and 55–60 min of recovery, respectively. All measurements and analyses were conducted by the same operator (YS), and only datasets achieving an operator index of 80% or higher were used in this study.

### Laser Doppler flowmetry

Red blood cell flux, an index of cutaneous vasodilation, was assessed by LDF (Nilsson et al. 1980). LDF probes were placed on the mid-ventral forearm and mid-ventral thigh, flush with the skin, to measure red blood cell flux. All participants were requested to remain as still as possible for 5 min before data recording. The raw LDF data recorded at 40 Hz were then averaged per second, and data points that deviated more than three standard deviations were excluded from analysis. All reported skin perfusion results were averaged over 30 s. Skin perfusion data were recorded at baseline, as well as at 20, 40, and 60 min during the intervention, and at 30 and 60 min during the recovery period. Furthermore, all flux values were converted to cutaneous vascular conductance (CVC) using the following formula: CVC = Flux/Mean arterial pressure.

### Blood analyses

Blood samples were collected using K_3_EDTA and lithium–heparin monovettes, and centrifuged at 3500 rpm for 10 min at 4 °C. The resulting plasma was aliquoted and stored at −80 °C pending batch analysis. K_3_EDTA plasma was used to analyse concentrations of 17β-estradiol (ALPCO, Salem, NH), progesterone (Abcam, Cambridge, UK), IL-6 (High Sensitivity, R&D Systems, Abingdon, UK), IL-1ra (Cytoscreen, Invitrogen, Paisley, UK), and IL-10 (High Sensitivity, Invitrogen, Paisley, UK) using enzyme-linked immunosorbent assays with intra-plate CVs of 2.5%, 2.4%, 6.0%, 7.0%, and 2.5%, respectively. Lithium–heparin plasma nitrite concentrations were determined by ozone chemiluminescence (Bailey et al. 2009). Initially, 500 μL of heparinised plasma was deproteinised by mixing with an equal volume of ice-cold ethanol and centrifuged at 2360 g for 10 min. The clear supernatant was then introduced into a gas-tight purge vessel via 200 μL injection through a septum. Nitric oxide (NO) was subsequently produced by reducing plasma nitrite in the presence of glacial acetic acid and 4% w/v aqueous sodium iodide solution. The chemiluminescence signal of NO reacting with ozone was recorded using a Sievers NOA 280i analyser (Analytix, Durham, UK), with the mV signal area being converted to concentration using a standard curve from nitrite standards across the nanomolar concentration range. Haematocrit and haemoglobin levels were measured in duplicate using a microcentrifuge and a Yumizen H500 automatic analyser (Horiba Medical, Montpellier, France). These measurements were used to adjust the concentrations of IL-6, IL-1ra, IL-10, and nitrite for changes in plasma volume (Dill and Costill 1974).

### Statistical analysis

All data are presented as mean ± SD. Normality and sphericity were checked using Shapiro–Wilk and Mauchly’s tests, respectively. Changes in physiological and thermoregulatory parameters and 17β-estradiol, progesterone, IL-6, IL-1ra, IL-10 and nitrite concentrations were analysed by a repeated measures analysis of variance (ANOVA) (Condition x Time x Sex), using pairwise comparisons when significant interactions were detected. Bonferroni-corrected post hoc pairwise comparisons were applied. Changes in perceptual measures were analysed non-parametrically. Friedman test was used to test for the effect of time in each condition, whereas Wilcoxon signed-rank tests were used for pairwise comparisons. Mann–Whitney *U* tests were performed to assess sex differences in perception scale data. The 29th version of SPSS (IBM Corp., Armonk, N.Y., USA) was used for all analyses, and significance was accepted at *p* ≤ 0.05.

## Results

Female participants had lower height and weight compared to male participants (*p* < 0.01), whilst their fat mass index, fat mass percentage, and skinfold thickness were higher than those of male participants (*p* ≤ 0.01; Table 1).

**Table 1:** Participant characteristics

	Conditions	Females (N = 10)	Males (N = 10)
Age (years)		26 ± 4	25 ± 3
Height (cm)^#^		165 ± 6	177 ± 5
Weight (kg)^#^		59 ± 6	72 ± 9
Fat mass (kg)		17.1 ± 2.7	13.9 ± 4.9
Fat mass index (kg/m^2^)^#^		6.3 ± 1.0	4.5 ± 1.5
Fat mass (% total body mass)^#^		29.1 ± 2.8	19.6 ± 5.0
Skinfold thickness (body fat%)^#^		27.0 ± 3.6	15.3 ± 3.3
Structured exercise (h/week)		5 ± 2	4 ± 3
Estradiol concentration (pg/ml)	CH	15.2 ± 5.7	14.9 ± 11.4
	IH	13.8 ± 6.3	15.0 ± 11.1
Progesterone concentration (ng/ml)	CH	0.6 ± 0.3	0.8 ± 0.4
	IH	0.5 ± 0.3	0.9 ± 0.4

All data are expressed as mean ± SD. 60 min continues heating (CH), 20 min × 3 intermittent heating (IH). Estradiol concentrations in one male and one female were below the lowest detectable 17β-estradiol concentration of 5.6 pg/mL in the IH condition. For statistical analysis, values below the lowest detectable 17β-estradiol concentration were assumed to be 5.6 pg/mL

^#^Significantly different between females and males (*p* ≤ 0.05)

### Temperature and sweat loss

**Fig. 2:**
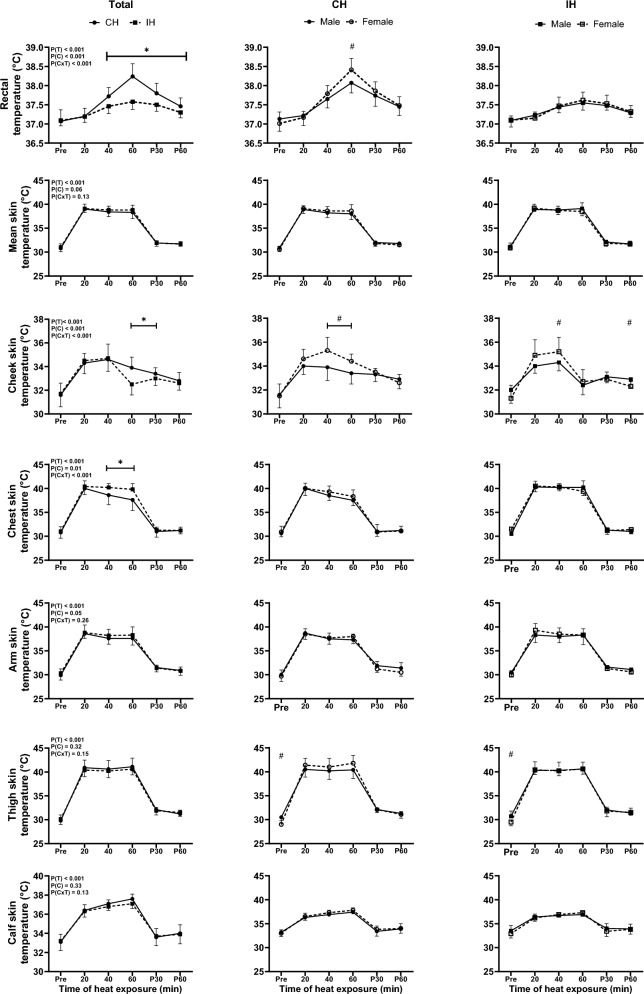
Acute changes in rectal, mean, cheek, chest, arm, thigh and calf skin temperature of all participants (Total), and separately for males and females for continuous heating (CH) and intermittent heating (IH). P(T), main effect of Time; P(C), main effect of Condition; P(CxT), Condition x Time interaction effect. Data are mean ± SD. *Difference between CH and IH, ^#^Difference between males and females (*p* ≤ 0.05). For panels comparing males and females, only the sex differences are reported

T_rec_ was elevated to a larger extent by CH compared with IH (Condition x Time, *p* < 0.001). At the end of CH, T_rec_ in females was higher than in males (*p* = 0.02). Mean T_skin_ during heat exposure showed no significant difference between CH and IH (Condition, *p* = 0.06; Time, *p* < 0.001). However, cheek T_skin_ was elevated to a greater extent by CH compared with IH (Condition x Time, p < 0.001). Cheek T_skin_ in females was higher compared to males at 40 min and 60 min of heat exposure in CH (*p* ≤ 0.01), and it was also higher in IH at 40 min (*p* = 0.05). However, cheek T_skin_ in females was lower compared to males in the recovery post-60 min of IH exposure (*p* = 0.03). The rise in chest T_skin_ was attenuated in CH compared with IH (Condition x Time, *p* < 0.001), with a mean increase of 7.1 ± 0.8℃ in CH and 8.8 ± 0.2℃ in IH. Similarly, the rise in arm T_skin_ was attenuated in CH compared with IH (Condition, *p*= 0.05; Time, *p* < 0.001), with mean increase of 7.8 ± 0.4℃ in CH and 8.1 ± 0.7℃ in IH. For thigh and calf T_skin_, there was a main effect of time (*p* < 0.001) with no difference between CH and IH (Condition, *p* = 0.32). In addition, thigh T_skin_ in females was lower compared to in males at baseline in both conditions (*p* = 0.01; Fig. 2).

T_rec_ iAUC in CH was larger than in IH during heat exposure (*p* = 0.01), with no sex difference (*p* = 0.27). Total body sweat loss was no different between CH and IH (*p* = 0.94), with no sex difference (*p* = 0.36). Arm and thigh sweat loss were both greater in CH compared to IH (*p* = 0.01 and *p* < 0.001, respectively). Arm and thigh sweat loss were lower in females than in males (*p* = 0.01 and *p* = 0.03, respectively; Table 2). For plasma volume, there was a Condition x Time interaction (*p* = 0.01). Plasma volume decreased in CH (pre: 59 ± 4%; post: 58 ± 5%; *p* = 0.02), but did not change in IH (pre: 58 ± 4%; post: 59 ± 5%; *p*= 0.42).

**Table 2:** Rectal temperature incremental area under curve and sweat losses

	Condition	Females (N = 10)	Males (N = 10)
**T**_**rec**_ incremental area under curve (℃·min)*	CH	33 ± 18	22 ± 9
	IH	13 ± 7	15 ± 10
Total sweat loss (ml)	CH	636 ± 143	763 ± 194
	IH	674 ± 357	714 ± 289
Arm sweat loss (ml)*	CH	0.35 ± 0.23	0.52 ± 0.28
	**IH**^**#**^	0.17 ± 0.10	0.38 ± 0.20
Thigh sweat loss (ml)*	CH	0.31 ± 0.13	0.46 ± 0.22
	**IH**^**#**^	0.22 ± 0.09	0.35 ± 0.13

All data are expressed as mean ± SD. Significant differences are highlighted in bold. 60 min continues heating (CH), 20 min × 3 intermittent heating (IH)

*Significantly different between CH and IH

^#^Significantly different between females and males (*p* ≤ 0.05)

### Skin perfusion

Arm and thigh skin perfusion increased in CH and IH during heat exposure (Condition, p ≥ 0.22; Time, p < 0.001; Condition x Time, *p* ≤ 0.04). Specifically, thigh skin perfusion was greater in CH than in IH at 60 min of heat exposure (*p* = 0.02), and both arm and thigh skin perfusion were higher in CH than IH at 30 min after exposure (*p* ≤ 0.05). In addition, thigh skin perfusion was lower in females than in males at 60 min post-exposure in the IH condition (*p* = 0.04).

**Fig. 3:**
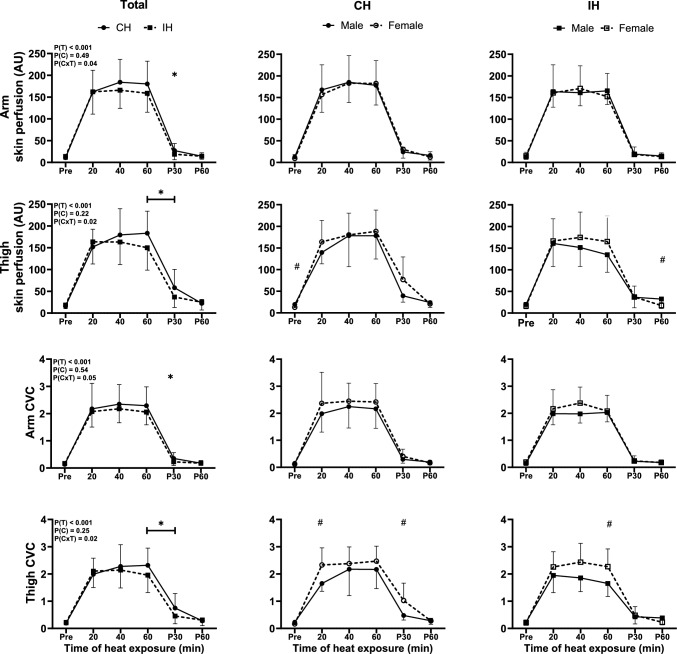
Arm and thigh skin perfusion and cutaneous vascular conductance (CVC) of all participants (Total), and separately for males and females for continuous heating (CH) and intermittent heating (IH). P(T), main effect of Time; P(C), main effect of Condition; P(CxT), Condition x Time interaction effect. Data are mean ± SD. *Difference between CH and IH, #Difference between males and females (*p* ≤ 0.05). For panels comparing males and females, only the sex differences are reported

Arm and thigh CVC increased in CH and IH during heat exposure (Condition, p ≥ 0.25; Time, p < 0.001; Condition x Time, *p* ≤ 0.05). Thigh CVC was higher in CH than IH at 60 min of heat exposure (*p* = 0.05), and both arm and thigh were higher in CH than IH at 30 min after exposure (*p* ≤ 0.04). In addition, thigh CVC in females was higher than in males at 20 min of heat exposure and at 30 min after exposure in CH (*p* ≤ 0.02), and at 60 min of heat exposure in IH (*p* = 0.03; Fig. 3).

### Brachial blood pressure and heart rate

The rise in brachial SBP was greater in CH compared with IH (Condition x Time, *p* < 0.001), but brachial DBP was decreased similarly following the heat exposure session in both CH and IH (Time, *p* < 0.001; Condition, *p* = 0.09; Condition x Time, *p* = 0.10). The brachial MAP decreased after heat exposure in CH and IH (Time, *p* = 0.01; Condition, *p* = 0.98; Condition x Time, *p* = 0.03), whilst brachial MAP in CH was lower compared with IH after 60-min heat exposure (*p* = 0.05). The rise in brachial PP was attenuated in IH compared with CH (Condition x Time, *p* < 0.001). Brachial SBP, DBP, MAP and PP in females were lower compared with males (*p* ≤ 0.01). The rise in HR was larger in CH compared with IH (Condition x Time, *p* < 0.001), with no sex difference in HR (*p* = 0.76; Table 3).

**Table 3:** Brachial blood pressure and heart rate response

	Total			Males			Females		
	Pre	Post	Post-60 min	Pre	Post	Post-60 min	Pre	Post	Post-60 min
SBP (mmHg)									
** CH**^**†#**^	109 ± 11	117 ± 12^*^	108 ± 11	117 ± 6	123 ± 9	114 ± 8	102 ± 9	111 ± 12	101 ± 10
** IH**^**†#**^	108 ± 11	110 ± 11^*^	108 ± 10	117 ± 5	116 ± 8	114 ± 8	100 ± 9	103 ± 11	101 ± 9
DBP (mmHg)									
** CH**^**†#**^	68 ± 4	61 ± 4	65 ± 5	71 ± 4	62 ± 5	67 ± 4	65 ± 3	59 ± 3	63 ± 5
** IH**^**†#**^	67 ± 6	62 ± 4	67 ± 4	70 ± 6	64 ± 4	69 ± 5	65 ± 6	59 ± 3	65 ± 3
MAP (mmHg)									
** CH**^**†#**^	80 ± 6	77 ± 5	77 ± 7^*^	84 ± 4	80 ± 5	80 ± 5	76 ± 4	75 ± 5	74 ± 6
** IH**^**†#**^	79 ± 7	77 ± 6	79 ± 5^*^	83 ± 6	80 ± 4	82 ± 5	75 ± 7	73 ± 5	76 ± 3
PP (mmHg)									
** CH**^**†#**^	41 ± 8	56 ± 12^*^	43 ± 8	46 ± 6	61 ± 11	47 ± 8	37 ± 7	52 ± 11	38 ± 7
** IH**^**†#**^	41 ± 9	48 ± 9^*^	41 ± 9	47 ± 7	51 ± 8	45 ± 9	35 ± 7	44 ± 9	36 ± 8
HR (beats/min)									
** CH**^**†**^	61 ± 11	98 ± 10^*^	63 ± 8	60 ± 9	100 ± 11	63 ± 9	62 ± 13	96 ± 10	63 ± 8
** IH**^**†**^	60 ± 7	85 ± 8^*^	62 ± 8	61 ± 6	84 ± 8	63 ± 8	59 ± 9	85 ± 9	60 ± 8

All data are expressed as mean ± SD. Significant differences are highlighted in bold. Brachial artery systolic blood pressure (SBP), diastolic blood pressure (DBP), mean arterial pressure (MAP), pulse pressure (PP) and heart rate (HR) at Pre, Post and Post-60 min in the 60 min continues heating (CH) and 20 min × 3 intermittent heating (IH)

^**†**^Effect of time

^#^Significantly different between females and males

^*^Significantly different between CH and IH (*p* ≤ 0.05)

### Central blood pressure and augmentation index

A Condition x Time interaction was observed for central SBP (*p* = 0.02), with CH exhibiting higher values than IH after 60 min of heat exposure (*p* = 0.01). Central DBP (Condition, *p* = 0.65; Time, *p* < 0.001; Condition x Time, *p* = 0.08) and MAP (Condition, *p* = 0.96; Time, *p* = 0.01; Condition x Time, *p* = 0.06) was decreased following the heat exposure session in CH and IH. Central PP was elevated to a larger extent by CH compared with IH (Condition x Time, *p* = 0.01). In addition, in females, central SBP, DBP, MAP and PP were lower than in males (*p* ≤ 0.01). AIx@HR75 was increased after the heat exposure sessions, and there was no difference between CH and IH (Condition, *p* = 0.57; Time, *p* < 0.001; Condition x Time, *p* = 0.61; Table 4).

**Table 4:** Central blood pressure and pulse wave analysis response

	Total			Males			Females		
	Pre	Post	Post-60 min	Pre	Post	Post-60 min	Pre	Post	Post-60 min
SBP (mmHg)									
** CH**^**#**^	93 ± 8	95 ± 8^*^	91 ± 8	99 ± 4	99 ± 6	96 ± 5	87 ± 6	91 ± 7	86 ± 8
** IH**^**#**^	93 ± 9	92 ± 7^*^	92 ± 7	99 ± 4	96 ± 4	96 ± 5	86 ± 8	88 ± 7	87 ± 6
DBP (mmHg)									
** CH**^**†#**^	69 ± 5	63 ± 5	66 ± 6	71 ± 4	66 ± 6	69 ± 5	66 ± 3	61 ± 3	63 ± 5
** IH**^**†#**^	68 ± 6	63 ± 4	68 ± 4	70 ± 6	66 ± 4	70 ± 5	65 ± 6	61 ± 3	66 ± 3
MAP (mmHg)									
** CH**^**†#**^	80 ± 6	77 ± 5	77 ± 7	84 ± 4	80 ± 5	81 ± 5	76 ± 4	75 ± 5	74 ± 6
** IH**^**†#**^	79 ± 7	76 ± 5	79 ± 5	83 ± 6	80 ± 4	82 ± 5	75 ± 7	73 ± 5	76 ± 4
PP (mmHg)									
** CH**^**†#**^	25 ± 5	31 ± 6^*^	25 ± 4	28 ± 3	33 ± 6	27 ± 4	22 ± 4	29 ± 5	23 ± 4
** IH**^**†#**^	25 ± 6	29 ± 5^*^	24 ± 5	29 ± 4	31 ± 4	26 ± 5	21 ± 4	27 ± 5	22 ± 5
AIx@HR75 (%)									
** CH**^**†**^	− 7 ± 10	8 ± 6	− 9 ± 9	− 9 ± 12	7 ± 5	− 12 ± 9	− 5 ± 7	10 ± 7	− 6 ± 9
** IH**^**†**^	− 8 ± 10	10 ± 8	− 9 ± 9	− 8 ± 12	7 ± 6	− 11 ± 11	− 8 ± 8	14 ± 7	− 6 ± 7

All data are expressed as mean ± SD. Significant differences are highlighted in bold. Central artery systolic blood pressure (SBP), diastolic blood pressure (DBP), mean arterial pressure (MAP), pulse pressure (PP), and AIx normalised for a heart rate of 75 beats per minute (AIx@HR75) heart rate of all participants (Total), and separately for males and females at Pre, Post and Post-60 min in the 60 min continues heating (CH) and 20 min × 3 intermittent heating (IH)

^**†**^Effect of time

*Significantly different between CH and IH

^#^Significantly different between females and males (*p* ≤ 0.05)

### Inflammatory and nitrite responses

**Fig. 4:**
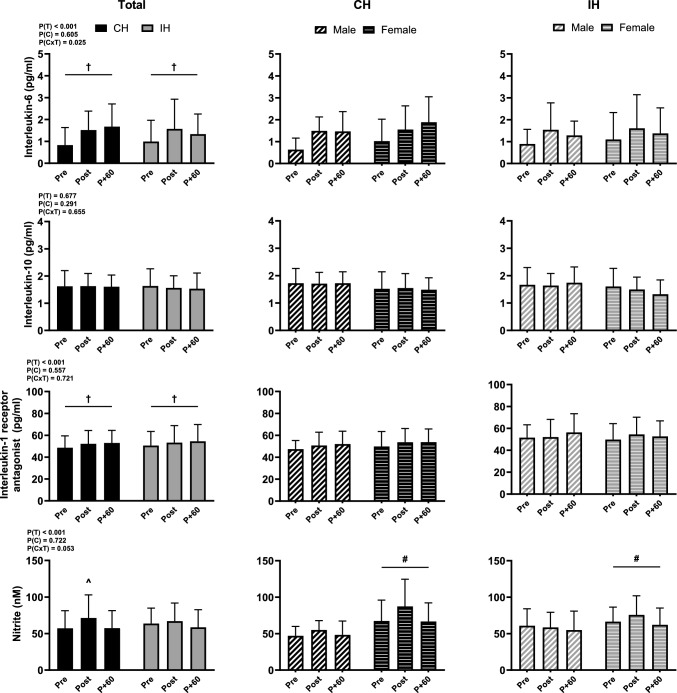
The interleukin-6, interleukin-10, interleukin-1 receptor antagonist and nitrite of all participants (Total), and separately for males and females for continuous heating (CH) and intermittent heating (IH). Data are mean ± SD. †Effect of time, ^Different from Pre, #Difference between males and females (*p* ≤ 0.05). For panels comparing males and females, only the sex differences are reported

Plasma IL-6 concentrations were elevated compared to pre in both conditions, with an interaction effect indicating a higher elevation in CH after 60-min heat exposure (Time, *p* < 0.001; Condition, *p* = 0.60, Condition x Time, *p* = 0.03). Plasma IL-10 concentrations were unaffected by Condition and Time (*p* ≥ 0.29), whilst plasma IL-1ra concentrations were increased (Time, *p* < 0.001). The plasma nitrite concentration was elevated after CH only (*p* < 0.001), with a significant Condition and Time interaction (*p* = 0.05). In addition, there was a main effect of sex in plasma nitrite concentration (*p* = 0.04; Fig. 4).

### Perceptual responses

**Fig. 5:**
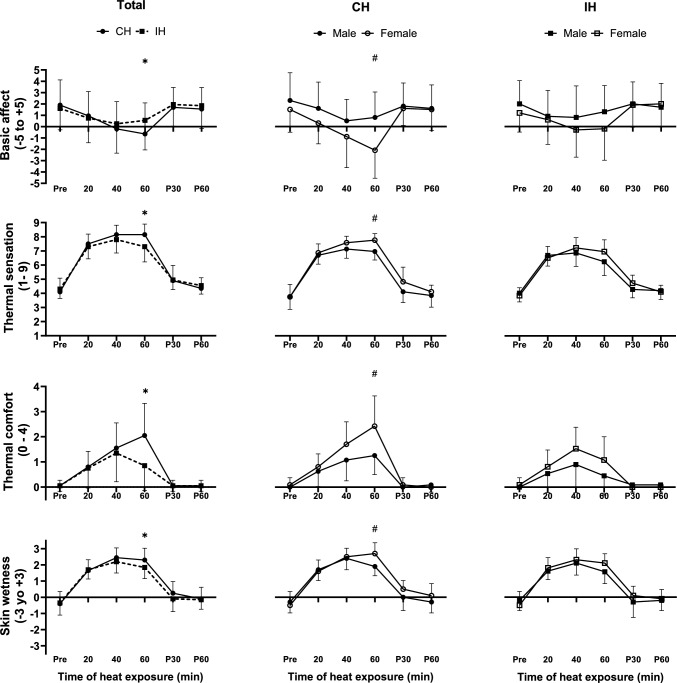
Basic affect, thermal sensation, thermal comfort and skin wetness in response to continuous heating (CH) and intermittent heating (IH) of all participants (Total), and separately for males and females for CH and IH. Data are mean ± SD. *Difference between CH and IH, #Difference between males and females (*p* ≤ 0.05). For panels comparing males and females, only the sex differences are reported

Basic affect, thermal sensation, and thermal comfort were all changed during both conditions (*p* < 0.001). Basic affect in CH was less favourable than in IH at 60 min of heat exposure (*p* = 0.01), and females experienced a less favourable basic affect than males at 60 min of CH (*p* = 0.02). Similarly, the thermal sensation in CH was higher than in IH at 60 min (*p* = 0.01), with females feeling hotter than males at 60 min of CH (*p* = 0.01). In addition, thermal comfort scores in CH were higher (= more uncomfortable) than in IH at 60 min (*p* < 0.001), and females felt more uncomfortable than males at 60 min of CH (*p* = 0.02). There was a main effect of time for skin wetness (*p* < 0.001). Furthermore, skin wetness in CH was higher than in IH at 60 min of heat exposure (*p* = 0.03) and was higher in females than males at 60 min of CH (*p* = 0.01; Fig. 5).

## Discussion

The main findings of this study were that (1) T_rec_ was greater in CH than in IH (2) Thigh CVC, SBP and the nitrite response were more pronounced in CH, whilst Aix@HR75 was not different between CH and IH. (3) Whilst the IL-6 response was blunted in IH, the acute elevation of IL-1ra was not different between CH and IH. (4) The perceptual response in IH was more favourable than in CH. (5) Cheek T_skin_, thigh CVC, and plasma nitrite concentration were higher, whilst brachial and central BP were lower in females than males in both conditions. However, a higher T_rec_ and a less favourable thermal perception in females were only observed in CH.

### Body temperature and thermal perception

#### Protocol comparison

Both T_rec_ and cheek T_skin_ during thermal exposure were higher in CH compared to IH, and T_rec_ iAUC was higher in CH than in IH. This is likely explained by the intermittent nature of the IH protocol, which included recovery cooling periods and mitigated the cumulative increase in body temperature despite the overall heat exposure being equal. Elevated core and T_skin_ increase thermal sensation and decrease thermal comfort (Chatonnet and Cabanac 1965; Frank et al. 1999). Moreover, changes in skin wetness have been shown to impact thermal comfort (Fukazawa and Havenith 2009). We found that the skin wetness perceptual response in CH was greater than in IH. Although overall sweat loss did not differ between protocols, local sweat loss in the arm and thigh was found to be greater in CH than in IH, which might explain the difference in wetness perception. Both findings on increased body temperatures and wetness perception, therefore, likely explain why IH was associated with improved basic affect and thermal comfort and reduced thermal sensation compared to CH. This suggests that IH represents a superior modality for those with lower heat tolerance.

#### Sex comparison

In CH, females had a more negative perceptual response, likely explained by their higher body temperature (T_rec_ and T_skin_) compared with males (Frank et al. 1999). Consistent with previous findings, the higher T_skin_ during passive heat exposure in females compared to males (Grucza et al. 1985) may be attributed to the higher percentage of body fat in females (Inoue et al. 2005) as the low thermal conductivity of fat enhances insulation and reduces heat transfer. In our study, females had approximately 12% higher body fat compared to males, which likely contributed to the observed differences. Moreover, the higher T_rec_ in females may be explained by their lower body mass and size than males, resulting in a lower body surface area and a higher body surface area to mass ratio, contributing to a less effective heat exchange (Havenith 2001). The lower body mass in females may hence explain the higher peak T_rec_ for the same heat exposure. The higher T_rec_ in females may further explain their higher cheek T_skin_, with blood directed to the facial area as a compensatory mechanism to dissipate heat (Ootsuka and Tanaka 2015). There was no sex difference in IH, indicating that the negative perceptions experienced in females during CH were mitigated by introducing breaks. Therefore, IH may specifically benefit females, as their higher fat mass and lower body mass predisposes them to greater risk of heat-related discomfort.

### Skin perfusion and nitrite response

#### Protocol comparison

The increase in arm and thigh skin perfusion and CVC was more pronounced in CH compared to IH. This is likely explained by the higher T_rec_ in CH than in IH, as this mediates vasodilation and enhances blood flow to the skin to dissipate heat (Chou and Coyle 2023). As NO contributes to vasodilation, the higher skin perfusion in CH may be mechanistically explained by the acutely elevated plasma nitrite concentrations (Kellogg et al. 1999). The more pronounced plasma nitrite response in CH found in the current study gives further evidence for a T_rec_ dose–response relationship for the NO response. Indeed, previous studies indicate that 45–60 min of whole body heat exposure leads to a T_rec_ increase of approximately 1.6℃, along with elevated concentrations of nitric oxide metabolites and plasma nitrite (Gryka et al. 2020; Hoekstra et al. 2021), which is similar to the rise we observed in T_rec_ (1.2℃) in CH. In contrast, others report no significant change in nitrite concentration after 60 min of lower limb heat exposure, with T_rec_ increasing by approximately 0.4 °C (Hoekstra et al. 2021), which is consistent with results observed in IH in the current study, in which T_rec_ increased by approximately 0.5°C. In summary, the cooling period of the IH protocol facilitated recovery and reduced the need for sustained systemic vasodilation. This may have inhibited the activation of endothelial NO synthase (Hodges et al. 2006), leading to reduced overall NO synthesis and its subsequent oxidation to nitrite.

#### Sex comparison

There was no sex difference for skin perfusion during the heat exposure period of CH and IH. However, females exhibited a higher thigh CVC compared to males. This observation may be explained by the higher plasma nitrite concentrations found in females in the present study, as increased nitrite concentrations can promote vasodilation and enhance CVC. Previous studies have shown that passive heat therapy improves cutaneous microvascular function through enhanced NO-dependent dilation (Brunt et al. 2016a). This NO-mediated vasodilation leads to decreases in blood pressure; the higher CVC observed can hence be attributed, in part, to the lower MAP we found in females.

### Blood pressure and augmentation index

#### Protocol comparison

Passive heat stress is associated with increased cardiac output and leads to peripheral vasodilation, which has been shown to increase SBP and decrease DBP (Chiesa et al. 2019). In line with this, we observed an increase in brachial SBP (but not central SBP) during heat exposure in CH. On the other hand, both brachial and central DBP decreased during heat exposure in CH and IH, with a reduced DBP being observed after heat exposure in CH. “Pressure wave amplification” is the process by which the arterial pressure wave increases in amplitude as it moves from the central arteries to the peripheral arteries (Agabiti-Rosei et al. 2007). Typically, the DBP changes little across the arterial tree, whereas SBP is amplified when moving from the aorta to the periphery (Nichols et al. 2022). Accordingly, we observed lower central SBP than brachial SBP both at rest and following heat exposure. Notably, after heating, there was a larger increase in HR, brachial and central SBP during CH compared to IH, whereas there was a larger decrease in brachial and central DBP after CH. The increase in HR is associated with increased sympathetic nervous system activation (Crandall et al. 2000). Moreover, thermal stress decreases peripheral resistance, thus requiring a higher cardiac output to maintain adequate perfusion (Kirsch et al. 1986) leading to increases in SBP. Persistent vasodilation during CH further decreases peripheral resistance, resulting in a more pronounced reduction in DBP. In contrast, the intermittent cooling periods of the IH protocol allowed peripheral resistance to recover periodically (Wilson et al. 2007), leading to a smaller blood pressure response compared to CH.

Correction for HR (i.e. assessing AIx@HR75) is crucial to examine heating-induced changes in wave reflection that are masked by HR (Wilkinson et al. 2000). We did indeed find HR in CH to be higher than IH at 60 min of heat exposure, correcting for this HR difference, we found no condition difference in AIx@HR75.

#### Sex comparison

In line with existing literature, both brachial and central BP were lower in females compared to males across the entire duration of CH and IH (Wiinberg et al. 1995). The BP difference has previously been attributed in part to the vasodilatory effects of estradiol, which increases endothelial NO production (Mendelsohn 2000). In the current study, estradiol concentrations did not differ between males and females, a likely effect of restricting data collection to the early follicular phase. However, females did exhibit higher NO concentration. The present study hence supports the mechanistic relationship between NO and blood pressure (Vallance and Chan 2001). Numerous factors may influence NO synthesis and regulation, including variations in muscle sympathetic nervous activity. It is known that sympathetic regulation of BP tends to be more pronounced in males than in females, which may contribute to differences in blood pressure regulation (Hart et al. 2009). Furthermore, the typically smaller hearts and lower stroke volume in women versus men is also thought to play a role (St. Pierre et al. 2022).

Estradiol may also influence arterial stiffness and contribute to variations in AIx@HR75; indeed, AIx@HR75 has shown to be lower when estradiol concentrations are high (Adkisson et al. 2010). Sex differences in Aix@HR75 may further arise due to its negative correlation with body height (Hayward and Kelly 1997; Rosenwasser et al. 2014) as a quicker return of the reflection wave from the periphery due to the reduced length of blood vessels (London et al. 1995). Despite this, no significant sex differences in AIx@HR75 were found in the present study, notwithstanding females in our cohort were of smaller stature than the males.

### Inflammatory response

#### Protocol comparison

Both protocols resulted in a significant increase in IL-6, evidencing that no substantial T_rec_ elevations are required for a systemic IL-6 response to occur (Hoekstra et al. 2021). Elevated IL-6 is a documented response to passive heating (Kuhlenhoelter et al. 2016; Brunt et al. 2018; Hoekstra et al. 2018), mediating the acute phase response and stimulating the release of anti-inflammatory cytokines such as IL-1ra and IL-10 (Petersen and Pedersen 2005). Whilst IL-1ra concentrations increased due to hyperthermia in the present study, evidence of the anti-inflammatory response, no changes in IL-10 concentrations were found after heat exposure. The lack of change in IL-10 concentrations suggests that neither passive heating protocol provided sufficient stress. A previous study reported elevated IL-10 concentrations in cancer patients when core temperatures reached 41.8 ℃ in induced hyperthermia (Robins et al. 1995), whilst no IL-10 changes were observed in ankylosing spondylitis patients and healthy control subjects when body core temperatures reached 38.7–39.0 ℃ (Zauner et al. 2014). Whilst inflammatory conditions may further impact the cytokine response, these data suggest that a more significant increase in core temperature may, therefore, be required to induce an IL-10 response.

#### Sex comparison

Estradiol can promote anti-inflammatory signals (Steiner and Berry 2022). However, we found no sex differences in IL-6, IL-10, and IL-1ra concentrations. Whether this is due to the similar estradiol concentrations between our male and female groups is unclear, yet others similarly report that IL-6, IL-10, and IL-1ra did not differ between males and females before and after a 2-h running experiment at 60% maximal oxygen uptake at 35 °C (Snipe and Costa 2018). Although the study by Snipe et al. (2018) did not report estradiol or progesterone concentrations, the experimental trial was completed between days 5 and 10 of the menstrual cycle, which is similar to the control of the menstrual cycle in our study. Taken together, Snipe et al. (2018) and our findings suggest that inflammatory responses to passive heating and prolonged exercise do not elicit sex differences in the inflammatory response when women are assessed during the low oestrogen phase of the menstrual cycle.

Further, a dose–response relationship has been suggested between elevated T_rec_ and acute IL-6 responses (Hoekstra et al. 2020). Whilst females (38.40 ± 0.30℃) reached significantly higher T_rec_ than males (38.07 ± 0.26℃) in CH, no difference in the IL-6 response was observed, implying that this T_rec_ difference was not pronounced enough to result in an altered inflammatory response.

## Limitations

This investigation exclusively focused on healthy and young males and females, which may restrict the generalisability of the findings to older adults or individuals with pre-existing health conditions. We further excluded highly trained individuals which may present with an altered inflammatory and thermoregulatory response due to training adaptations. Consequently, the cardiovascular responses to passive heating observed in this cohort may differ in more diverse populations. Whilst we measured a range of cardiovascular and inflammatory variables, the assessment of pulse wave velocity, flow-mediated dilation, and heart rate variability could shed more light on mechanistic relationships. Furthermore, a limitation of this study is the lack of an a-priori sample size calculation. The sample size was informed by a previous study that examined sex differences in skin blood flow response to passive heating involving data from ten young women and six young men (Inoue et al. 2005). However, the reported metrics were insufficient to calculate the sample size for the interactions that were the primary focus of this study.

## Conclusion

Including breaks into a passive heating protocol increases the tolerability to heat exposure. Acute increases in cardiovascular and inflammatory variables were found for both heating protocols, but the inclusion of breaks blunted the increase of some of them. These findings may be particularly relevant to females and other individuals with lower heat tolerance.

## Data Availability

Data will be made available upon request.

## Abbreviations

AIx@HR75: Augmentation index adjusted for heart rate of 75 bpm
ANOVA: Analysis of variance
CH: Continuous heating
CVC: Cutaneous vascular conductance
DBP: Diastolic blood pressure
HR: Heart rate
iAUC: Incremental area under curve
IH: Intermittent heating
IL-10: Interleukin-10
IL-1ra: Interleukin-1 receptor antagonist
IL-6: Interleukin-6
LDF: Laser Doppler flowmetry
MAP: Mean arterial pressure
NO: Nitric oxide
PP: Pulse pressure
SBP: Systolic blood pressure
T_rec_: Rectal temperature
T_skin_: Skin temperature
